# Quercetin downregulates Mcl-1 by acting on mRNA stability and protein degradation

**DOI:** 10.1038/bjc.2011.229

**Published:** 2011-07-12

**Authors:** C Spagnuolo, C Cerella, M Russo, S Chateauvieux, M Diederich, G L Russo

**Affiliations:** 1 Institute of Food Sciences, National Research Council, 83100 Avellino 83100 Avellino, Italy; 2 Laboratoire de Biologie Moléculaire et Cellulaire du Cancer, Fondation de Recherche sur le Cancer et les Maladies du Sang, Hôpital Kirchberg, L-2540 Luxembourg, Luxembourg

**Keywords:** chronic lymphocytic leukaemia, apoptosis, Mcl-1, quercetin, gossypol, chemotherapy

## Abstract

**Background:**

We recently demonstrated that quercetin, a flavonoid naturally present in food and beverages belonging to the large class of phytochemicals, was able to sensitise leukaemic cells isolated from patients with chronic lymphocytic leukaemia (CLL) when associated with recombinant tumour necrosis factor-related apoptosis-inducing ligand (TRAIL) or anti-CD95. We also showed that quercetin potentiated the effect of fludarabine on resistant B cells from CLL patients. Resistance to therapy in CLL depends on the expression and activity of anti-apoptotic proteins of the Bcl-2 family. Among these, myeloid cell leukaemia-1 (Mcl-1) has been associated with apoptotic resistance in CLL. Therefore, we investigate here whether the sensitising activity of this flavonoid, which leads to increased apoptosis in both cell lines and CLL, could be related to Mcl-1 expression and stability.

**Results:**

B cells isolated from CLL patients showed different levels of Mcl-1 protein expression, resulting, in several cases, in increased sensitivity to fludarabine. Quercetin significantly enhanced the downregulation of Mcl-1 in B cells isolated from selected patients expressing detectable levels of Mcl-1. In U-937 cells, quercetin increased Mcl-1 mRNA instability in the presence of actinomycin D. When cells were treated with MG-132, a proteasome inhibitor, Mcl-1 protein level increased. However, quercetin, in the presence of Z-Vad-FMK, continued to lower Mcl-1 protein expression, indicating its independence from caspase-mediated degradation. In contrast, co-treatment of quercetin and MG-132 did not revert the effect of MG-132 mono-treatment, thus suggesting a possible interference of quercetin in regulating the proteasome-dependent degradation of Mcl-1. Gossypol, a small-molecule inhibitor of Bcl-2 family members, mimics the activity of quercetin by lowering Mcl-1 expression and sensitising U-937 cells to apoptosis induced by recombinant TRAIL and the Fas-ligand.

**Conclusion:**

This study demonstrates that in U-937 cells, quercetin downregulates Mcl-1 acting directly or indirectly on its mRNA stability and protein degradation, suggesting that the same mechanism may bypass resistance to apoptosis in leukaemic cells isolated from CLL patients and sensitise B cells to apoptosis induced by drugs and death receptor inducers.

Chronic lymphocytic leukaemia (CLL) is the most frequent form of leukaemia in adults in the Western world with >12 000 cases yearly in the United States (Pekarsky *et al*, 2010). It is characterised by a progressive accumulation of small, mature B cells with typical B-cell markers, such as CD19, CD23 and CD20, as well as inappropriate expression of the T-cell antigen, CD5 (Chiorazzi *et al*, 2005; Chen and Plunkett, 2010). Some patients with CLL survive for many years or decades without any treatment because of the relatively slow progression rate of the disease. Other patients experience a rapid and fatal disease despite therapy. Treatment depends on the clinical staging, Rai or Binet, which classify patients according to tumour burden and haematopoietic impairment (Rai *et al*, 1975; Binet *et al*, 1981). Therapy available for the treatment of CLL includes chemotherapy with agents such as chlorambucil, cyclophosphamide, fludarabine and bendamustine (Hallek *et al*, 2010; Hertlein and Byrd, 2010). Fludarabine generated a significant improvement in responses compared with alkylating agents (Rai *et al*, 2000). Moreover, randomised trials with combinations of fludarabine and cyclophosphamide generated better results than did treatments by fludarabine alone (Flinn *et al*, 2007). As a result, 25–40% patients gain a complete response to DNA-directed agents. However, despite the improved efficacy of CLL treatment, relapse is frequent. More recently, treatment with humanised monoclonal antibodies, such as rituximab and alemtuzumab has been used as an induction therapy for refractory CLL (Yee and O’Brien, 2006; Tam *et al*, 2008). Very recently, the German Chronic Lymphocytic Leukaemia Study Group initiated a phase 3 trial to compare the efficacy and safety of fludarabine and cyclophosphamide *vs* fludarabine, cyclophosphamide and rituximab as first-line treatment in patients with advanced, symptomatic chronic lymphocytic leukaemia. In the chemoimmunotherapy group, 65% of patients were free of progression compared with 45% in the chemotherapy group. In addition, chemoimmunotherapy improves overall survival in patients with CLL (Hallek *et al*, 2010). Nevertheless, as relapse remains problematic, particularly in older patients, the identification of innovative and specific therapies for CLL remains of high interest (Chen and Plunkett, 2010). In fact, a significant percentage of untreated patients (up to 37%) does not respond to fludarabine treatment and up to 76% of patients become refractory to this regime of treatment (Yee and O’Brien, 2006).

The presence of anti-apoptotic proteins of the Bcl-2 family (Gottardi *et al*, 1996) is an important mechanism involved in CLL resistance to therapy. Overexpression of the anti-apoptotic B-cell lymphoma (Bcl-2) protein is common in CLL (Pepper *et al*, 1997, 1998) because of hypomethylation of the promoter region of the *Bcl-2* gene (Hanada *et al*, 1993; Pepper *et al*, 2008) or because of a lack of expression of miR-15a and miR-16-1, which regulate Bcl-2 at the posttranscriptional level (Cimmino *et al*, 2005). In addition, high levels of Bcl-2 have been associated with shorter overall survival in previously treated patients and increased chemoresistance to treatment with fludarabine (Robertson *et al*, 1996). It is worthwhile to note that an important determinant in CLL is the relative expression of Bcl-2 compared with pro-apoptotic Bax. In fact, increased Bcl-2/Bax ratio in CLL is associated with the progressive pattern of disease (Molica *et al*, 1998; Pepper *et al*, 1998). More recently, another Bcl-2 family member, myeloid cell leukaemia sequence (Mcl)-1, has been associated with apoptotic resistance in CLL (Smit *et al*, 2007; Longo *et al*, 2008). Low expression of Mcl-1 mRNA is correlated with prolonged survival in B-cell CLL (Veronese *et al*, 2008).

Myeloid cell leukaemia-1 downregulation induces apoptosis in a number of leukaemia-derived cell lines and enhances rituximab-mediated apoptosis (Derenne *et al*, 2002; Michels *et al*, 2004; Hussain *et al*, 2007). In primary B cells isolated from patients with CLL, Mcl-1 protein expression has been shown to correlate with an adverse prognosis if combined with other prognostic markers, such as the stage of the disease, IgV_H_ mutation status, ZAP-70 positivity and CD38 expression (Pepper *et al*, 2008). In CLL patients treated with pentostatin, cyclophosphamide and rituximab showing high expression of Mcl-1, both minimal residual disease-negative status and progression-free survival were found to be significantly reduced (Awan *et al*, 2009). Therefore, Mcl-1 expression may be useful in predicting poor response to chemoimmunotherapy.

Myeloid cell leukaemia-1 was discovered as a pro-survival member of the Bcl-2 family rapidly responding to phorbol 12-myristate 13-acetate-induced differentiation of myeloid leukaemia cells (Kozopas *et al*, 1993). The carboxy-terminal region of the protein contains three putative BH domains, which mediate its anti-apoptotic function. In fact, Mcl-1 binds and sequesters pro-apoptotic Bax and Bak blocking their ability to form pores in the mitochondrial membrane and to release cytochrome *c* into the cytoplasm. Degradation of Mcl-1 frees Bax and Bak allowing their polymerisation and activating apoptosis (Thomas *et al*, 2010). Myeloid cell leukaemia-1 shows a very short half-life of mRNA and protein (Yang *et al*, 1995; Schubert and Duronio, 2001), and the regulation of its expression can occur at multiple levels: (1) several transcription factors can regulate Mcl-1 transcription; (2) an alternative splicing produces two isoforms with opposite functions; (3) microRNAs (miRNAs) and RNA-binding proteins are responsible for translational control; (4) degradation depends on caspase-mediated and/or proteasome-dependent mechanisms; (5) post-translational regulation involves phosphorylation/de-phosphorylation events on different Mcl-1 residues leading to enhancing or inhibiting Mcl-1 apoptotic features (Akgul, 2009; Thomas *et al*, 2010).

These data suggest that knowledge of the post-translational modifications leading to increased or decreased half-life of the Mcl-1 protein in B cells may lead to important therapeutic applications in CLL, as well as in other forms of cancer. As an example, the multi-kinase inhibitor sorafenib, recently approved for the treatment of renal cancer and currently undergoing clinical trials for a wide range of human cancers, downregulates Mcl-1 and cellular inhibitor of apoptosis-2 expression sensitising cancer cells to tumour necrosis factor (TNF)-related apoptosis-inducing ligand (TRAIL)-induced cell death (Ricci *et al*, 2007). Similarly, suppression of Mcl-1 expression in some leukaemias and lymphomas potentiates vinblastin-induced apoptosis (Salerni *et al*, 2010).

To overcome resistance in cancer therapy, a plethora of naturally occurring molecules with chemopreventive properties has been suggested as potential candidates in adjuvant chemotherapy when associated with other drugs (Aggarwal and Shishodia, 2006; Mansour *et al*, 2007; Russo, 2007; Fimognari *et al*, 2008; Reuter *et al*, 2008). One of these compounds, quercetin (3,3′,4′,5,7-pentahydroxyflavone), a naturally occurring flavonoid widely present in fruits and beverages (Lamson and Brignall, 2000; Day *et al*, 2003), attracted our attention as this molecule was able to re-establish sensitivity to apoptosis induction in leukaemic cell lines resistant to CD95- and TRAIL-induced cell death (Russo *et al*, 1999, 2003, 2007). Very recently, we demonstrated that quercetin was able to sensitise leukaemic cells isolated from CLL patients when associated with recombinant TRAIL (rTRAIL) or anti-CD95 (Russo *et al*, 2010). We also showed that quercetin potentiated the effect of fludarabine on resistant B cells from CLL patients (Russo *et al*, 2010).

In this study, we demonstrated that quercetin is able to lower the expression of Mcl-1 acting on several regulatory steps. This effect can be associated with the ability of the molecule to sensitise U-937 cells to apoptosis triggered by fludarabine and death receptor inducers reported previously (Russo *et al*, 2007).

## Materials and methods

### Reagents

Roswell Park Medium Institute (RPMI) medium, L-glutamine 200 mM, penicillin 5000 IU ml^−1^/streptomycin 5000 *μ*g ml^−1^ and phosphate-buffered saline (PBS) tablets were purchased from Invitrogen (S. Giuliano Milanese, Italy). Neutral red 0.33% solution, propidium iodide, trypan blue solution (0.4%), quercetin, gossypol, Hoechst 33342 and dimethyl sulfoxide (DMSO) were purchased from Sigma-Aldrich (Milan, Italy). Recombinant TRAIL (super killer TRAIL) was from Enzo Life Sciences (AG Lausen, Switzerland). The Fas ligand (Fas-L) was obtained from Millipore (Brussels, Belgium). Fludarabine phosphate (F) was kindly donated by the Onco-Haematology Division (S.G. Moscati Hospital, Avellino, Italy).

### Cell isolation and viability tests

Mononuclear cells (leukaemic cells >90%) were isolated from peripheral blood of patients affected by CLL. All clinical samples were obtained with informed consent. After density gradient centrifugation (Ficoll-Paque Plus, GE Healthcare, Milan, Italy), cells were washed three times in PBS, counted with trypan blue dye to assess their viability (cell viability >95%) and immediately cultured in RPMI supplemented with 1% penicillin/streptomycin, 2 mM L-glutamine and 10% autologous serum (Bomstein *et al*, 2003), at 37°C in a humidified atmosphere containing 5% CO_2_. For neutral red assays (Fautz *et al*, 1991), cells were cultured at a density of 1 × 10^6^ per ml in 48-well plates and incubated (24–48 h) in a medium containing 0.1% DMSO, 10–25 *μ*M quercetin solubilised in 0.1% DMSO or fludarabine dissolved in PBS (3.5 *μ*M final concentration). Cell viability assay was performed as described previously (Russo *et al*, 2003).

The human myelomonocytic cell line U-937 was purchased from Deutsche Sammlung von Mikroorganismen und Zellkulturen GmbH (Braunschweig, Germany). Cells were cultured in complete RPMI 1640 medium (Lonza, Verviers, Belgium) supplemented with 10% (v/v) fetal bovine serum (Lonza) and 1% (v/v) penicillin/streptomycin (BioWhittaker, Verviers, Belgium) at 37°C in a humidified atmosphere containing 5% CO_2_. Treatments included incubation at indicated times with DMSO (control), quercetin 25 *μ*M, transcription inhibitor actinomycin D (5 *μ*g ml^−1^), proteasome inhibitor MG-132 (5 *μ*M) (Sigma-Aldrich) and the general caspase inhibitor Z-Vad-FMK (10 *μ*M) (BD Pharmigen, Milan, Italy).

### Apoptotic assays

U-937 cells were treated with 25 *μ*M quercetin, 10 *μ*M gossypol, 5 ng ml^−1^ rTRAIL, 50 ng ml^−1^ Fas-L and their associations for 16 h. To assess induction of apoptosis, two different assays were used: reduction of mitochondrial membrane potential by MitoTracker Red CMXRos (Invitrogen) and staining with the DNA-specific dye Hoechst 33342. In the first case, U-937 cells were incubated for 20 min at 37°C in the presence of 50 nM MitoTracker Red according to the manufacturer's protocol before flow-cytometric analysis (FACSCalibur; BD Biosciences, San Jose, CA, USA). In the case of Hoechst 33342 staining, percentages of apoptotic cells, quantified as the fraction of apoptotic nuclei, were assessed by fluorescence microscopy (Leica-DM IRB microscope; Leica, Lecuit, Luxembourg) upon dye addition at the final concentration of 1 *μ*g ml^−1^. At least 300 cells in three independent fields were counted to evaluate the presence of nuclei with apoptotic morphology.

### Immunoblotting

Expression of Bcl-2 (Calbiochem Merck Chemicals Ltd, Nottingham, UK), Mcl-1 and *β*-actin (Cell Signaling, Milan, Italy) in CLL cells was revealed by immunoblotting using specific antibodies as described previously (Russo *et al*, 2007). Chronic lymphocytic leukaemia cells (2 × 10^6^ per ml) were suspended in lysis buffer containing 150 mM NaCl, 50 mM Tris-HCl, pH 7.4, 5 mM ethylenediaminetetraacetic acid, 1% NP-40, 0.5 mM dithiotreitol, 1 mM Na_3_VO_4_, 40 mM NaF, 1 mM Na_4_P_2_O_7_, 7.4 mg ml^−1^ 4-*p*-nitrophenyl phosphate, 10% glycerol, 100 *μ*g ml^−1^ phenylmethylsulfonyl fluoride and a protease inhibitor cocktail (Complete; Roche, Monza, Italy). Total protein lysates (20–25 *μ*g) were loaded on a 12% pre-cast gel (CRITERION XT, Bio-Rad Laboratories, Segrate, Milan, Italy) and blotted onto polyvinylidene difluoride (PVDF), Hybond-P membrane (GE Healthcare). The membrane blots were rinsed with T-TBS (0.1% Tween-20, 25 mM Tris, 137 mM NaCl, 2.69 mM KCl, pH 8) and blocked by 5% (w/v) non-fat dry milk in T-TBS for 1 h at room temperature. The membrane was then incubated for 16 h at 4°C with specific antibodies. The PVDF membrane was finally incubated with horseradish peroxidase-linked secondary antibody against mouse (GE Healthcare). The immunoblots were developed using Western Lightning Chemiluminescence Reagent Plus (Perkin-Elmer, Monza, Italy).

In the case of U-937 cell line, after indicated treatments, cells were lysed using M-PER (mammalian protein extraction reagent) (Pierce, Erembodegem, Belgium) according to the manufacturer's instructions. In brief, 4 × 10^6^ cells per sample were washed with PBS and the pellet was re-suspended in 250 *μ*l of M-PER containing 40 *μ*l ml^−1^ protease inhibitor cocktail (Complete; Roche, Prophac, Luxembourg), 1 mM Na_3_VO_4_, 5 mM NaF, 1 mM PMSF and 100 *μ*l ml^−1^ phosphatase inhibitor PhosSTOP (Roche). The suspension was clarified by vertical agitation for 15 min at 4°C, followed by a centrifugation at 15 000 × **g** for 15 min. Total protein extracts (20 *μ*g) were loaded on 12% SDS–PAGE, transferred onto PVDF membranes and blocked with 5% non-fat milk in PBS-Tween 0.1% for 1 h at room temperature. Blots were incubated with primary antibodies: anti-Mcl-1 (Cell Signaling, Bioké, Leiden, The Netherlands) and anti-*β*-actin (Sigma-Aldrich, Bornem, Belgium) according to the provider's protocols. After incubation with primary antibodies, membranes were incubated with the corresponding secondary, and specific immunoreactive proteins were visualised by autoradiography using the ECL Plus Western Blotting Detection System Kit (GE Healthcare, Diegem, Belgium). Luminescence signal was acquired using ImageQuant LAS 4000 mini (GE Healthcare), and the optical density of bands was evaluated on Gel Doc 2000 (Bio-Rad Laboratories) and analysed using the Multi-Analyst Software (Bio-Rad Laboratories).

### RNA extraction, RT and real-time PCR quantification

U-937 cells were treated, lysed and total RNA was extracted using Trizol Reagent (Invitrogen, Life Technologies, San Giuliano Milanese, Italy) according to the manufacturer's protocol. RNA quantification was assessed by Nanodrop (Isogen Life Science, Sint-Pieters-Leeuw, Belgium). Reverse transcription (RT) for cDNA synthesis was performed on 3 *μ*g total RNA using the SuperScriptTM III first-strand synthesis system (Invitrogen) and random hexamer primers. Real-time PCR analysis was performed using the SYBR Green PCR Master Mix (Applied Biosystems, Monza, Italy) according to the manufacturer's protocol with a 7300 Real-Time PCR System (Applied Biosystems, Lennik, Belgium). Quantification was performed in triplicate, and expression levels of Mcl-1 (forward-CCAAGGCATGCTTCGGAAA, reverse-TCACAATCCTGCCCCAGTTT) were normalised using internal standards: *β*-actin (forward-CCAAGGCATGCTTCGGAAA, reverse-TCACAATCCTGCCCCAGTTT). Relative gene expression levels correspond to fold induction (2^−ΔΔCt^) compared with untreated cells. Significant differences were determined using Student's *t*-test. Statistical significances were evaluated at *P*<0.05.

## Results

**Figure 1:**
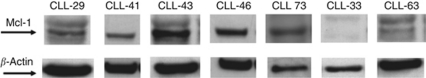
Mcl-1 expression in B cells isolated from CLL patients. B cells were isolated as reported in the ‘Materials and methods’ section. After cell lysis and immunoblotting, membranes were incubated for 16 h at 4°C with anti-Mcl-1 polyclonal antibody. In all cases, membranes were re-probed with an anti *β*-actin polyclonal antibody. Images are representative of one experiment out of two performed for each sample.

**Figure 2:**
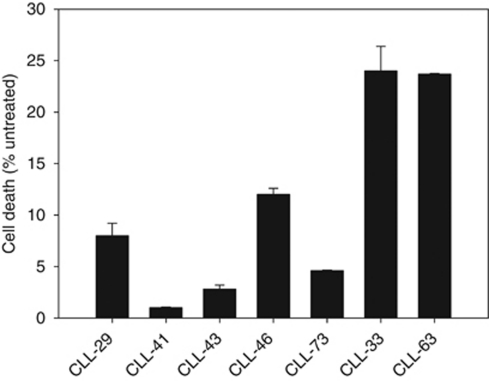
Cell viability assay in cells isolated from CLL patients and treated for 48 h with fludarabine at a concentration of 3.5 *μ*M. Cytotoxicity was measured by neutral red assay. Values are presented as mean of triplicate samples±s.e.m. compared with DMSO-treated cells.

**Figure 3:**
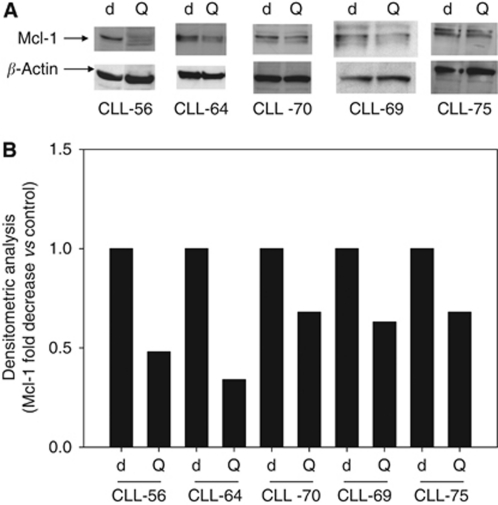
Downregulation of Mcl-1 expression by quercetin in B cells isolated from CLL patients. (**A**) In selected samples (CLL-56, CLL-64, CLL-69, CLL-70, CLL-75), B cells were isolated as reported in the ‘Materials and methods’ section and treated with 0.1% DMSO (d), or 10-20 *μ*M quercetin (Q). After cell lysis and immunoblotting, membranes were incubated for 16 h at 4°C with anti-Mcl-1 polyclonal antibody. In all cases, membranes were re-probed with an anti *β*-actin polyclonal antibody. Images are representative of one experiment out of two performed for each sample. (**B**) Band intensities were quantified measuring optical density on Gel Doc 2000 and analysed by Multi-Analyst Software.

We recently demonstrated that resistance to death receptor- and fludarabine-induced cell death in leukaemic cells isolated from CLL patients can be improved or bypassed by the combined treatment with quercetin (Russo *et al*, 2010). To investigate the mechanism(s) of action possibly triggered by quercetin, we tested its ability to interfere with the regulation of Bcl-2 protein expression. The expression levels of pro-apoptotic Bax and anti-apoptotic Bcl-xL showed strong fluctuations in B-CLL and were not sensitive to quercetin treatment ((Russo *et al*, 2010) and data not shown). B-cell lymphoma expression appeared more constant in B-CLL isolated from patients, but it was not influenced by quercetin treatment ((Russo *et al*, 2010) and Supplementary Figure S1)). Therefore, considering the important role of Mcl-1 in resistance to chemotherapy in CLL (Awan *et al*, 2009), we measured the ability of quercetin to regulate Mcl-1 expression. According to previous publications (Vogler *et al*, 2009), not all B-CLL cells expressed Mcl-1 (Figure 1). In our screening, ∼60% of samples showed detectable amounts of Mcl-1 (data not shown). Considering that Mcl-1 is phosphorylated by different kinases (Akgul, 2009; Thomas *et al*, 2010), we attributed *bona fide* the upper band observed in few samples in Figure 1 to phosphorylation form(s) of the protein. In agreement with the role of Mcl-1 in chemotherapeutic resistance, we observed that in two selected samples showing low or undetectable levels of Mcl-1 (CLL-33 and CLL-63 in Figure 1), fludarabine was more effective in inducing cell death as measured by neutral red assay (Figure 2), while samples with detectable levels of Mcl-1 were resistant to cell death induced by fludarabine (Figure 2). Therefore, we selected B cells isolated from five CLL patients expressing significant levels of Mcl-1 and treated them with selected concentrations of quercetin (10–20 *μ*M), which did not generate any cytotoxicity (data not shown). In all cases reported (Figures 3A and B), treatment with quercetin downregulated Mcl-1 expression, suggesting that quercetin may interfere with Mcl-1 stability at a transcriptional and/or translational level.

**Figure 4:**
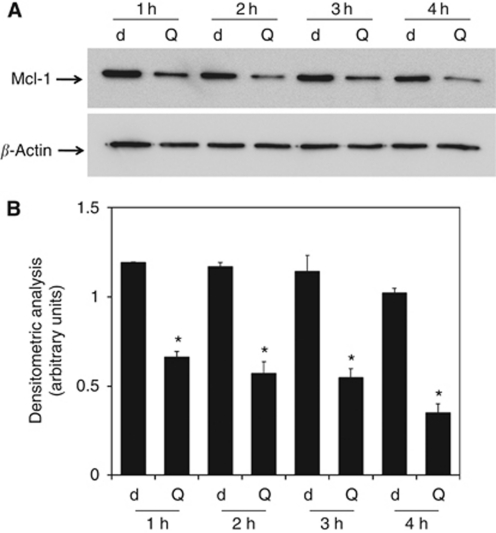
Mcl-1 protein expression in U-937 cells treated with quercetin. (**A**) Cells were treated with 0.1% DMSO (d) and 25 *μ*M quercetin (Q) for designed periods. After cell lysis and immunoblotting, membranes were incubated for 16 h at 4°C with anti-Mcl-1 polyclonal antibody. In all cases, membranes were re-probed with an anti *β*-actin polyclonal antibody. Two additional experiments yielded similar results. (**B**) Band intensities were quantified measuring optical density on Gel Doc 2000 and analysed by Multi-Analyst Software. Values in bar graphs represent means±s.e.m. for three separate experiments performed. Asterisks indicate significant difference from untreated U-937 (^*^P<0.005).

To explore this hypothesis, we used U-937 cells derived from a human monocytic leukaemia and expressing high levels of Mcl-1. We previously demonstrated that this cell line was resistant to anti-CD95- and TRAIL-induced apoptosis and that quercetin was able to enhance apoptotic response in the presence of those death ligands (Russo *et al*, 2007). First, we confirmed the downregulation of Mcl-1 after treatment with 25 *μ*M quercetin in U-937 (Figures 4A and B). At this concentration, the molecule was not cytotoxic and enhanced apoptosis induced by death ligands (rTRAIL and anti-CD95 antibody) as reported previously (Russo *et al*, 2007). In Figure 4A, we stimulated U-937 cells for 1–4 h and observed a significant decrease in Mcl-1 expression after quercetin treatment. Maximal downregulation was detectable at 4 h, as evidenced by densitometric analysis (Figure 4B). We also calculated the half-life of Mcl-1 as previously determined by others in different cell lines (Adams and Cooper, 2007). In U-937, Mcl-1 was degraded with a half-life of ∼30 min, after inhibition of protein synthesis (Supplementary Figure S2).

**Figure 5:**
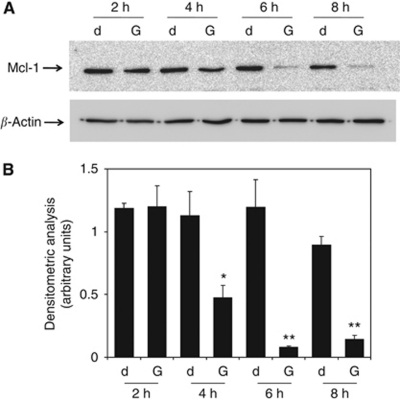
Mcl-1 protein expression in U-937 cells treated with gossypol. (**A**) Cells were treated with 0.1% DMSO (d) and 10 *μ*M gossypol (G) for indicated times; after cell lysis and immunoblotting, membranes were incubated for 16 h at 4°C in the presence of anti-Mcl-1 and anti *β*-actin polyclonal antibodies. (**B**) Band intensities were quantified measuring optical density on Gel Doc 2000 and analysed by Multi-Analyst Software. Values in bar graphs represent means±s.e.m. for three separate experiments performed. Asterisks indicate significant difference from untreated U-937 cells (^*^P<0.05; ^**^P<0.005).

**Figure 6:**
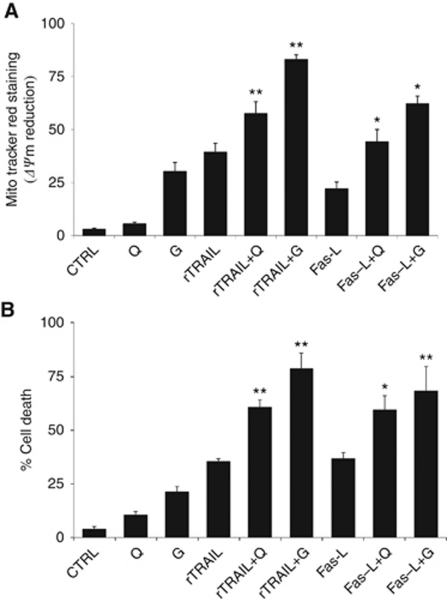
Effects of quercetin and gossypol on cell death. (**A**) U-937 cells were treated with 0.1% DMSO (CTRL), 25 *μ*M (Q) quercetin, 10 *μ*M (G) gossypol, 5 ng ml^−1^ rTRAIL, 50 ng ml^−1^ Fas-L and their associations for 16 h. Induction of apoptosis was evaluated by quantifying the percentage of cells presenting a reduction of mitochondrial membrane potential by MitoTracker Red CMX Ros as reported in the ‘Materials and methods’ section. (**B**) Treatments were carried out as described above, whereas the percentage of apoptotic cells was calculated by staining with the DNA-specific dye Hoechst 33342, followed by fluorescence microscopy assessment. At least 300 cells in 3 independent fields were counted to evaluate the presence of nuclei with apoptotic morphology. Values in bar graphs represent means±s.e.m. for four separate experiments performed. In combined treatments, apoptosis increased significantly with respect to the stimulation with single death ligand as indicated by asterisks (^*^P<0.05; ^**^P<0.005).

To strengthen the importance of Mcl-1 as the target of quercetin to sensitise cells to apoptosis, we demonstrated that a similar effect could be obtained following a quercetin-independent targeting of Mcl-1 accomplished by substituting the molecule with gossypol (2,2′-bis(8-formyl-1,6,7-trihydroxy-5-isopropyl-3- methylnaphthalene),C_30_H_30_O_8_) (Mohammad *et al*, 2005), a small-molecule inhibitors of Mcl-1 (Azmi and Mohammad, 2009). This compound is a polyphenol extracted from cottonseeds and roots. Previous reports have indicated that the anti-cancer effect of gossypol was due to its ability to interfere with the functions of Mcl-1, Bcl-2 and Bcl-xL (highest to lowest affinity) proteins (Mohammad *et al*, 2005; Etxebarria *et al*, 2008; Meng *et al*, 2008). Recently, a gossypol enantiomer, AT-101 has been shown to induce apoptosis in B-CLL cells and overcome stromal cell-mediated Mcl-1 induction and drug resistance (Balakrishnan *et al*, 2009). Figure 5 shows that gossypol reduced Mcl-1 protein expression similarly to quercetin (Figure 4), but at later time points (50% decrease of Mcl-1 starting from 4 h of treatment compared with 1–2 h for quercetin). At the tested concentration (10 *μ*M) and for the indicated length of treatment, gossypol, like quercetin, did not significantly decrease Bcl-2 and Bcl-xL protein levels (Supplementary Figure S3) without inducing apoptosis on U-937 (Supplementary Figure S4). Decreasing Mcl-1 protein levels was not sufficient to trigger *per se* the apoptotic machinery, but it could be an important target to sensitise cells to death. In fact, when quercetin or gossypol was associated with apoptotic inducers, such as the death ligands rTRAIL or Fas-L, we observed a significant increase in cell death compared with mono-treatments (Figure 6). This effect was confirmed by two independent but complementary assays to estimate apoptotic cells, such as the reduction of mitochondrial membrane potential (Figure 6A) and the presence of apoptotic nuclei (Figure 6B).

**Figure 7:**
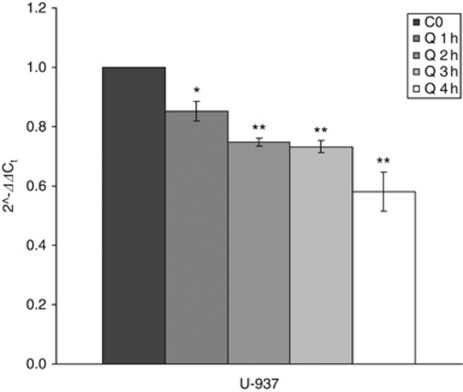
Mcl-1 mRNA expression in U-937 cell lines. Cells were treated with 0.1% DMSO (d) and 25 *μ*M quercetin (Q) for designed intervals, after which total RNA were isolated and Mcl-1 mRNA were quantified using real-time PCR as described in the ‘Materials and methods’ section. Values represent means±s.e.m. for three separate experiments performed in triplicate and relative quantification was performed using the Rq method 2^−ΔΔCt^. Asterisks indicate significant difference from untreated U-937 cells (^*^P<0.05; ^**^P<0.005).

**Figure 8:**
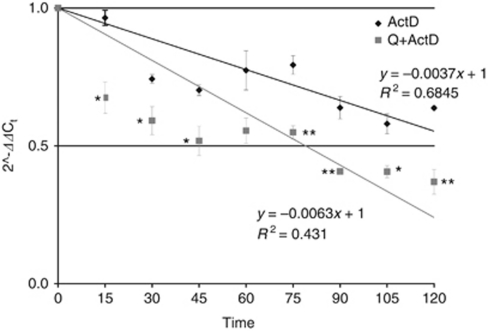
Mcl-1 mRNA stability in U-937 cell line. Cells were pre-treated for 1 h with 25 *μ*M quercetin (Q) and stimulated with 5 *μ*g ml^−1^ actinomycin D (ActD) at intervals of 15 min for 2 h. After stimulation, total RNAs were isolated and Mcl-1 mRNA was quantified using qPCR as described in the ‘Materials and methods’ section. Values represent means±s.e.m. for three separate experiments performed in triplicate and relative quantification was performed using the Rq method 2^−ΔΔCt^. Asterisks indicate significant difference from ActD-treated U-937 cells (^*^P<0.05; ^**^P<0.005).

As Mcl-1 can be regulated at multiple levels, the reduced protein expression could be explained by transcriptional or post-transcriptional inhibition, or by both. Therefore, we first investigated the ability of quercetin to modulate Mcl-1 mRNA expression by qPCR. As shown in Figure 7, quercetin significantly reduced mRNA levels in a time-dependent manner, which paralleled with Mcl-1 protein decrease (Figure 4), suggesting a regulation at the transcriptional level or an effect on mRNA stability. We tested the latter hypothesis, assessing Mcl-1 mRNA stability in the presence of quercetin using actinomycin D, a well-known inhibitor of transcription. In U-937 cells, actinomycin D decreased Mcl-1 mRNA by ∼50% in 135 min, whereas the association with quercetin accelerated mRNA reduction to 79 min (Figure 8), indicating the ability of the molecule to interfere with one, or more processes regulating mRNA stability.

**Figure 9:**
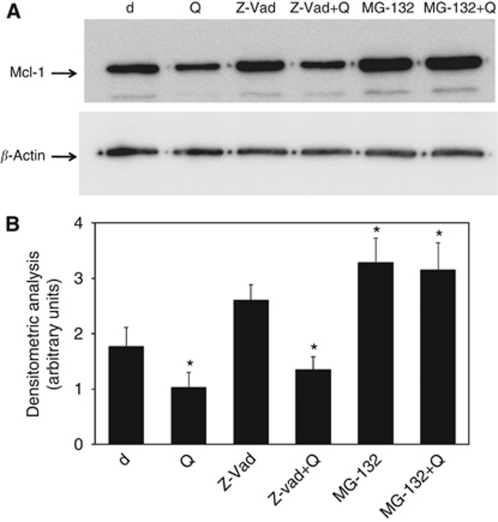
Mcl-1 protein degradation in U-937 cell lines. (**A**) Cells were pre-treated for 1 h with the caspase inhibitor Z-Vad-FMK (Z-Vad; 10 *μ*M) and MG-132 (5 *μ*M) before quercetin (Q) addition (25 *μ*M) for 2 h. After cell lysis and immunoblotting, membranes were incubated 16 h at 4°C with anti-Mcl-1 polyclonal antibody. In all cases, membranes were re-probed with an anti *β*-actin polyclonal antibody. (**B**) Band intensities were quantified measuring optical density on Gel Doc 2000 and analysed by Multi-Analyst Software. Values in bar graph represent means±s.e.m. for three separate experiments performed. Asterisk indicates significant differences with respect to untreated U-937 cells (^*^P<0.005).

Myeloid cell leukaemia-1 protein degradation is mediated by proteasome- and/or caspase-dependent mechanisms. Both processes rapidly decrease its cellular level (Michels *et al*, 2004; Zhong *et al*, 2005). To investigate whether quercetin could be also implicated in this regulation, we treated U-937 with Z-Vad-FMK, a caspase inhibitor and MG-132, a proteasome inhibitor, in the presence of quercetin. Immunoblots (Figure 9A) and densitometric analysis (Figure 9B) show that only MG-132 efficiently increased Mcl-1 protein level. However, quercetin, in the presence of Z-Vad-FMK, further decreased Mcl-1 protein level, indicating its independence from caspase-mediated degradation. On the contrary, co-treatment of quercetin together with MG-132 did not revert the effect of MG-132 mono-treatment, suggesting that quercetin may directly or indirectly interfere with proteasome-dependent degradation of Mcl-1 in addition to the already observed destabilisation of the corresponding mRNA.

## Discussion

Myeloid cell leukaemia-1 anti-apoptotic activity generates drug resistance and it is regulated at different levels. More specifically, its mRNA and protein stability represents a key control step in leukaemia. An Mcl-1 transgenic mouse model allowed to demonstrate that elevated Mcl-1 levels generate haematopoietic cells refractory to chemotherapy and perturb lymphopoiesis (Campbell *et al*, 2010). Therefore, the identification of compounds that decrease Mcl-1 protein levels is of potential therapeutic interest. Both Mcl-1 mRNA and protein have relatively short half-lives. This feature is exploited in cancer therapy, as inhibition of transcription and/or translation can rapidly diminish Mcl-1 levels in cells the survival of which mainly relies on Mcl-1 expression.

In this study, we hypothesised, for the first time to our knowledge, how quercetin, a naturally occurring flavonoid known for its ability to sensitise leukaemic cells to apoptotic inducers (fludarabine and death receptor inducers) can induce Mcl-1 downregulation, described in both U-937 cells and primary B cells. Our data suggest novel activity of the molecule on two regulatory levels: translation and transcription of Mcl-1. The pleiotropic nature of quercetin allows modulation of multiple cellular processes acting simultaneously on different cellular targets, which regulate cell death and cell growth (Russo, 2007). This behaviour seems to be confirmed in U-937 cells in which the molecule downregulates Mcl-1 expression triggering multiple regulatory pathways. To support and strengthen the conclusion of this work, very recently, it has been reported that quercetin induced apoptosis in U-937 through a process involving Mcl-1 downregulation (Cheng *et al*, 2010). These authors also showed that silencing Mcl-1 expression by siRNA and overexpressing the DN mutant of Mcl-1 enhanced and counteracted the apoptotic effects of quercetin (Cheng *et al*, 2010). The conclusions of the study by Cheng *et al* and those of this study complement each other. In fact, the former demonstrates the quercetin, at high concentration, induces apoptosis by reducing Mcl-1 expression, while we report that, at lower concentrations, quercetin does not induce apoptosis *per se*, but sensitises cells to apoptosis triggered by drugs (such as fludarabine) or death receptors inducers (Fas-L, rTRAIL) by decreasing Mcl-1 stability. The latter effect may be relevant in combined therapeutic protocols. In fact, Mcl-1 represents an ideal target for different and complementary therapeutic approaches acting directly or indirectly on its turnover. As an example, seliciclib, a cyclin-dependent kinase inhibitor, induced rapid dephosphorylation of the carboxyl-terminal domain of the large subunit of RNA polymerase II. Phosphorylation at these sites is crucial for RNA polymerase II-dependent transcription resulting in rapid degradation of Mcl-1 (MacCallum *et al*, 2005). Sorafenib (BAY 43-9006, a multi-kinase inhibitor) has been also shown to enhance Mcl-1 downregulation in actinomycin D-treated cells, suggesting attenuation of Mcl-1 translation in association with the rapid and potent dephosphorylation of the eukaryotic initiation factor-4E (eIF4E) translation-initiation factor. Sorafenib also sensitises resistant hepatocellular carcinoma cells (HCCs) to TRAIL-induced apoptosis in TRAIL-resistant HCCs by downregulation of phosphorylated signal transducer and activator of transcription (STAT)3 (pSTAT3) and subsequently reduced the expression levels of STAT3-related proteins, such as Mcl-1, survivin and cyclin D1 (Chen *et al*, 2010). Finally, the small BH3 mimetic obatoclax (GX015-070), which can bind the BH3-binding groove of the Bcl-2 family proteins and neutralise anti-apoptotic proteins, is able to interfere with the direct interaction between Mcl-1 and Bak, inducing cell death together with other mechanisms (Nguyen *et al*, 2007; Trudel *et al*, 2007). These published data support the concept that a molecule, able to act at different regulatory levels, finds in Mcl-1 an ideal substrate considering the complex, inter-connected and redundant pathways focusing on Mcl-1 regulation (Thomas *et al*, 2010). We suggest that quercetin can be a part of this group of compounds.

In this study, we show that treatment with quercetin destabilises Mcl-1 mRNA in U-937 cells. How this happens at the molecular level is actually under investigation. We already verified that quercetin does not interfere with the expression of a sample of five miRNAs, which target Mcl-1 (Supplementary Figure S5). Therefore, we hypothesise here the interference of quercetin in two processes regulating Mcl-1 at the translational level. First, it is known that Mcl-1 can be regulated by RNA-binding proteins, such as CUGBP2, which binds to 3′-untranslated region (UTR) of Mcl-1 mRNA and inhibits its translation (Subramaniam *et al*, 2008), or HuR (Abdelmohsen *et al*, 2007), which also binds to and promotes the expression of Mcl-1 mRNA. At least in the case of RNA-binding protein of the Hu family (HuR), in different systems, the accumulation and activity of this RNA-binding protein is under the control of protein kinase, such as p38 (Farooq *et al*, 2009), cJun N-terminal kinase (Hostetter *et al*, 2008), extracellular signal-regulated kinase (ERK) (Yang *et al*, 2004) and AKT (Kang *et al*, 2008). As the ability of quercetin to inhibit several serine-threonine kinases (Hou and Kumamoto, 2010) including phosphatidylinositol 3-kinase (Hwang *et al*, 2009) and mitogen-activated protein kinase ERK kinase-1 (Lee *et al*, 2008), we can postulate that the molecule could participate in Mcl-1 mRNA stability acting on key kinases regulating the different pathways controlling Mcl-1 expression at the translational level. To confirm this possibility, it has been recently reported that quercetin strongly inhibits binding of HuR to the AU-rich element in the Mcl-1 3′UTR to stabilise TNF-*α* mRNA (Chae *et al*, 2009). Furthermore, eIF4E has an important role in regulating translation initiation and in Mcl-1 stability. The kinase inhibitor BAY 43-9006 was shown to induce apoptosis in human leukaemia cells involving downregulation of Mcl-1 through the rapid and potent dephosphorylation of the eIF4E translation-initiation factor (Rahmani *et al*, 2005). It could be of interest to investigate the possible role of quercetin in this pathway. In fact, quercetin, among other compounds, increases the susceptibility of cervical carcinoma cells to CD40L-induced apoptosis reversing CD40-mediated dissociation of the translational repressor eIF4E-binding protein from the initiation factor eIF4E (Hill *et al*, 2005).

The N-terminal region (residues 1–170) is specific of Mcl-1; it is not homologous to Bcl-2 and it may contribute to protein-specific functions. This region contains four PEST sequences (namely P, proline; E, glutamic acid; S, serine and T, threonine), which act in general as signal peptides for protein degradation and are common features of unstable proteins (Kozopas *et al*, 1993; Thomas *et al*, 2010). In addition, the N-terminal region contains three ubiquitination sites and, within the PEST regions, two caspase cleavage sites are present together with several phosphorylation sites, which act as double-sided swords, being able to enhance or inhibit Mcl-1 apoptotic features (Thomas *et al*, 2010). Quercetin interferes positively with the proteasome-dependent degradation of Mcl-1 in U-937 (Figure 9) and in Jurkat T cells (data not shown) with unknown mechanisms. We can hypothesise different modes of action, which need to be experimentally proven. However, it is worthwhile to note that the existence of a functional relationship between quercetin and other flavonoids with proteasome regulation is not new. As an example, in malignant glioma cells, TRAIL-induced apoptosis was enhanced by quercetin-induced proteasomal degradation of survivin (Siegelin *et al*, 2009). In addition, quercetin-induced polyubiquitination of Her-2/neu, the elevated expression level of which is associated with poor prognosis in breast cancer, decreasing its protein level in a time- and dose-dependent manner (Jeong *et al*, 2008). However, these data contrast with other results suggesting that flavonoids might act as proteasome inhibitors decreasing cancer risk in tumours in which proteasome activity is required for cancer cell survival (Chen *et al*, 2005; Chang, 2009). Moreover, in a recent paper, Liu *et al* (2008) demonstrated that quercetin was able to inhibit the effect of bortezomib in CLL patients because of its ability to bind to the boronic acid group of that molecule.

From data presented in Figures 1 and 2, we can hypothesise that resistance to apoptogenic stimuli (death receptors inducers, fludarabine, other chemotherapic drugs) is blocked by increased expression levels of Mcl-1 in leukaemic cells isolated from CLL patients. We postulated that this resistance might be bypassed or improved by a treatment with quercetin, which decreases Mcl-1 protein level. However, it is worthwhile to note that quercetin *per se* is not able to induce apoptosis even if Mcl-1 is downregulated. In fact, as reported recently by our laboratory (Russo *et al*, 2010), quercetin activity results in the capacity to sensitise B cells and cell lines to apoptotic inducers, rather than to kill them. We hypothesised that quercetin may lower the threshold of resistance to apoptotic drugs in leukaemic cells acting at a different level and taking advantage of its pleiotropic activities. To support this view, we reported here that gossypol, a small molecule known for its ability to inhibit Mcl-1 expression, behaved similarly to quercetin; for example, at low concentration, it was able to decrease Mcl-1 expression without any effect on apoptosis (Supplementary Figure S4). Only when associated with death receptors inducers, the ability of gossypol to lower Mcl-1 expression resulted in enhanced apoptosis (Figure 6).

Chronic lymphocytic leukaemia is a heterogeneous leukaemia and the progressive resistance of patients to conventional treatments hinders improved therapies. Our study opens new applicative perspectives supported by the observation that specific targets for Mcl-1 in leukaemia are actively studied. A treatment in which quercetin would be combined to one or more Mcl-1 inhibitors may result in a significant improvement of therapy in both CLL and other types of cancer.
