# Supplementary material for: Quercetin downregulates Mcl-1 by acting on mRNA stability and protein degradation
Source: Br J Cancer. 2011 Jul 12;105(2):221–30. doi: 10.1038/bjc.2011.229 (PMC3142809; doi:10.1038/bjc.2011.229)
Supplement: Supplementary Information [file bjc2011229x1.doc]

**Manuscript: TH/2010/4324 (422)**

***Supplementary material***

**Quercetin down-regulates Mcl-1 by acting on mRNA stability and protein degradation**

Carmela Spagnuolo, Claudia Cerella, Maria Russo, Sébastien Chateauvieux, Marc Diederich and Gian Luigi Russo

**
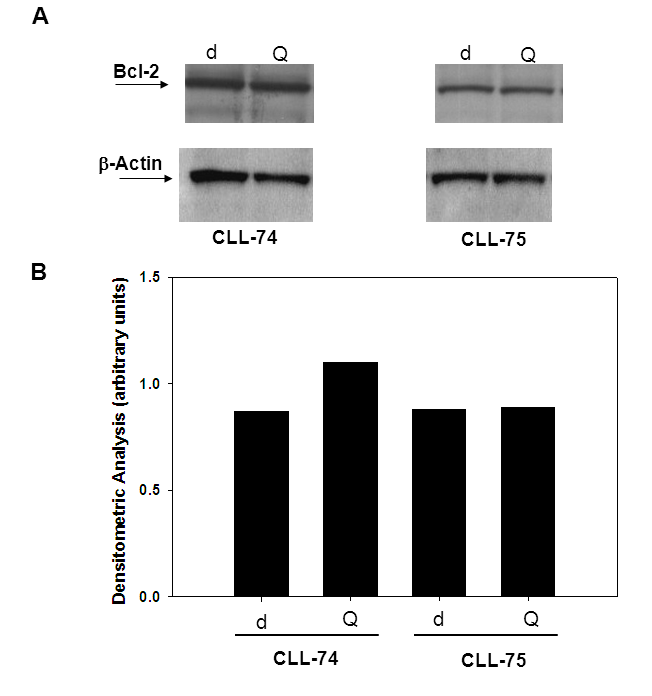
**

**Figure S1.** Bcl-2 expression in B-cells isolated from CLL patients. **A.** In CLL-74 and CLL-75 samples, cells were treated with 0.1% DMSO (d), or 20 μM quercetin (Q). After cell lysis and immunoblotting, membranes were incubated 16 h at 4°C with anti-Bcl-2 polyclonal antibody (Calbiochem Merck Chemicals Ltd; Nottingham, UK). In all cases, membranes were re-probed with an anti β-actin polyclonal antibody. **B.** Band intensities were quantified measuring optical density on a Gel Doc 2000 (Biorad) and analyzed by Multi-Analyst Software (Biorad).

Images are representative of one experiment out of two performed for each sample.

**
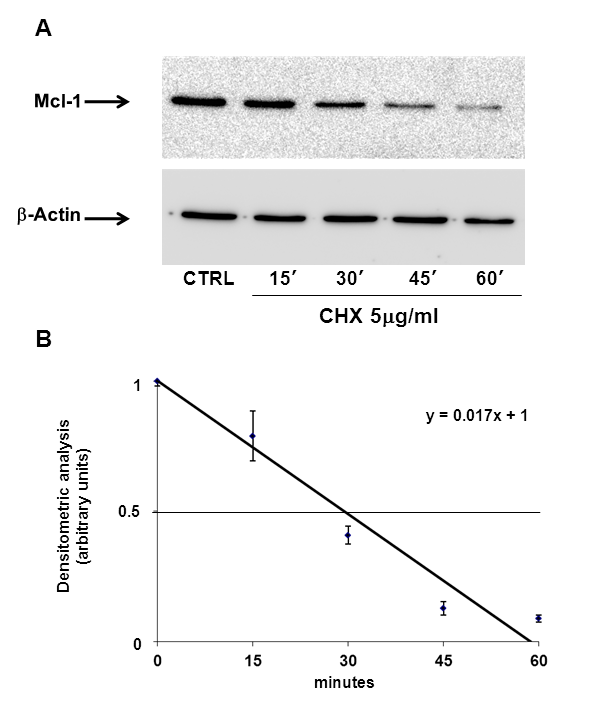
**

**Figure S2.** Mcl-1 protein half-life in U-937 cell line. **A.** To measure the half-life of Mcl-1 protein in U-937 cells, a time-course in the presence of 5 μg/ml cycloheximide (CHX; Sigma-Aldrich) was performed. After cell lysis and immunoblotting, membranes were incubated 16 h at 4°C in the presence of anti-Mcl-1 and anti β-actin polyclonal antibodies. Band intensities were quantified using a Gel Doc 2000 and analyzed by Multi-Analyst Software. **B.** Half-life of Mcl-1 protein was calculated by plotting in ordinate optical density (arbitrary unit) calculated from densitometric analysis of bands in panel A versus minutes of treatment (abscissa). Data in the graph represent the means ± sem for three separate experiments. Half-life of Mcl-1 protein is of 29.41 min.


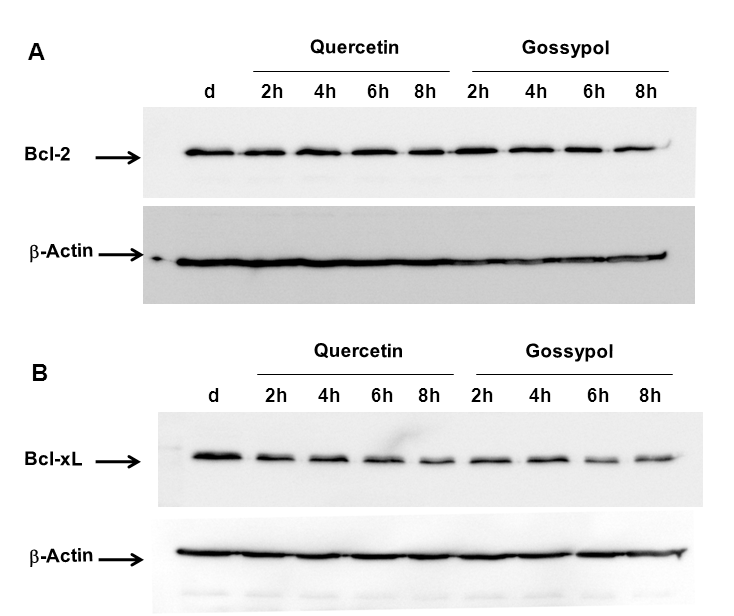


**Figure S3.** Bcl-2 and Bcl-xL protein expression in U-937 cells treated with quercetin and gossypol. U-937 cells were treated with 0.1% DMSO (d), 25 μM quercetin (Q) or 10 μM gossypol (G) for the indicated time. After cell lysis and immunoblotting, membranes were incubated 16 h at 4°C with anti-Bcl-2 monoclonal antibody (Calbiochem, Leuven, Belgium). **(A)** and anti-Bcl-xL polyclonal antibody (Transduction Laboratories, Erembodegem, Belgium). **(B)**. In both cases, membranes were also probed with an anti β-actin polyclonal antibody. Images are representative of one experiment out of two performed for each sample.

Neither quercetin, nor gossypol decreased significantly Bcl-2 and Bcl-xL protein expression.

**
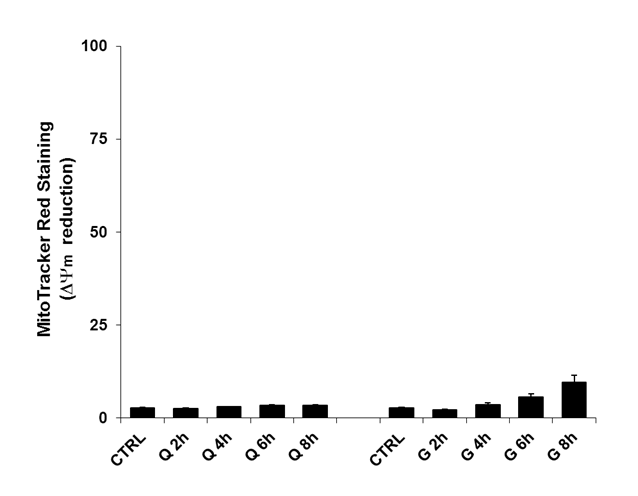
**

**Figure S4.** Effects of quercetin and gossypol on cell death. U-937 cells were treated with quercetin 25 μM (Q) or gossypol 10 μM (G) for indicated times and stained with 50 nM MitoTracker Red CMX Ros for 20 min at 37ºC accordingly to the manufacturer’s protocol before flow cytometric analysis. Values in bar graphs represent the means ± sem of three separate experiments performed.

Quercetin and gossypol did not significantly induce apoptosis in U-937 cells.


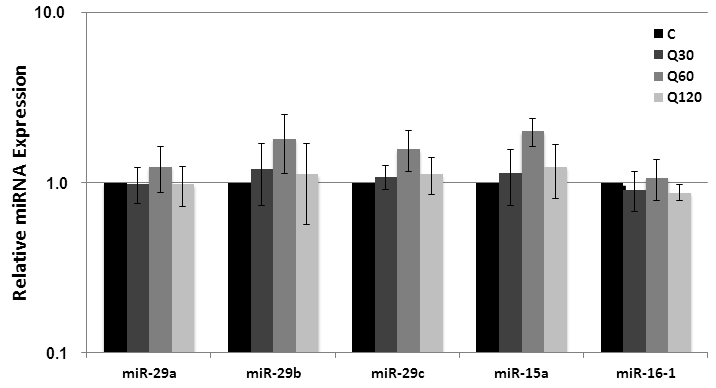


**Figure S5.** miRNA analysis in U-937 cells treated with quercetin. Total RNA from U-937 cells treated with 25μM quercetin for indicated times, was extracted using Trizol reagent (Invitrogen, Merelbeke, Belgium) and RNA quantified by spectrophotometry (Nanodrop® ND-1000) according to the manufacturer’s protocol. Reverse transcription of miRNAs were performed using the miScript Reverse Transcription Kit (Qiagen, Venlo, The Netherlands), starting from 1 μg of total RNA. PCR amplification were performed with miScript SYBR Green PCR Kit (Qiagen), according to the manufacturer’s protocol, with primers designed by Qiagen and described elsewhere : Hs_RNU1A_1 (MS00013986), Hs_miR-15a_1 (MS00003178), Hs_miR-16_1 (MS00006517), Hs_miR-29a_1 (MS00003262), Hs_miR-29b_1 (MS00006566) and Hs_miR-29c_1 (MS00003269). The samples were analyzed in a 7300 Real Time PCR system (Applied Biosystems, Halle, Belgium). The results were calculated with the ΔΔCT method and were normalized with the Hs_RNU1A_1 as reference. C: control; Q30: Quercetin 30 minutes, Q60: Quercetin 60 minutes, Q120: Quercetin 120 minutes.

Data indicate that quercetin does not significantly interfere with the expression of miRNAs able to target Mcl-1

**References in supplementary material**

Aqeilan RI, Calin GA, Croce CM (2010) miR-15a and miR-16-1 in cancer: discovery, function and future perspectives. *Cell Death Differ* **17**(2): 215-20

Desjobert C, Renalier MH, Bergalet J, Dejean E, Joseph N, Kruczynski A, Soulier J, Espinos E, Meggetto F, Cavaille J, Delsol G, Lamant L (2011) MiR-29a downregulation in ALK-positive anaplastic large-cell lymphomas contributes to apoptosis blockade through MCL-1 overexpression. *Blood* [Epub ahead of print]

Mott JL, Kobayashi S, Bronk SF, Gores GJ (2007) mir-29 regulates Mcl-1 protein expression and apoptosis. *Oncogene* **26**(42): 6133-40

Mraz M, Pospisilova S, Malinova K, Slapak I, Mayer J (2009) MicroRNAs in chronic lymphocytic leukemia pathogenesis and disease subtypes. *Leuk Lymphoma* **50**(3): 506-9

Pekarsky Y, Zanesi N, Croce CM (2010) Molecular basis of CLL. *Semin Cancer Biol* **20**(6): 370-6
